# Flood preparedness and public health policy, Greece

**DOI:** 10.2471/BLT.25.293793

**Published:** 2026-02-09

**Authors:** Varvara A Mouchtouri, Zacharoula Bogogiannidou, Ioanna Voulgaridi, Maria Kyritsi, Sotirios Vasileiadis, Athanasios G Lianos, Christos Kontos, Vasileios Nakoulas, Efthymia Petinaki, Kassiani Mellou, Kassiani Gkolfinopoulou, Theologia Sideroglou, Danai Pervanidou, Antonios Michaelakis, Eleni Patsoula, Annita Vakali, George Koliopoulos, Nikos T Papadopoulos, John Vontas, Georgia D Mandilara, Maria Tseroni, Dimitrios Paraskevis, Kyriaki Tryfinopoulou, Ioanna Spiliopoulou, Georgios Dougas, Eva Aggelouli, Dionysia Mintza, Fani Miskaki, Lemonia Anagnostopoulos, Fani Kalala, Matthaios Speletas, Dimitrios Hatzigeorgiou, Eirini Agapidaki, Christos Hadjichristodoulou

**Affiliations:** a Laboratory of Hygiene and Epidemiology, Faculty of Medicine, University of Thessaly, Papakyriazi 22, Larissa, 41222, Greece.; b Department of Microbiology, General University Hospital of Larissa, Larissa, Greece; c General Directorate of Epidemiological Surveillance, National Public Health Organization, Athens, Greece.; d Scientific Directorate of Entomology and Agricultural Zoology, Benaki Phytopathological Institute, Athens, Greece.; e School of Public Health, University of West Attica, Athens, Greece.; f School of Plant Sciences, Agricultural University of Athens, Athens, Greece.; g Department of Agriculture, Crop Production and Rural Environment, University of Thessaly, Volos, Greece.; h Pesticide Science Lab, Agricultural University of Athens, Athens, Greece.; i Department of Nursing, National and Kapodistrian University of Athens, Athens, Greece.; j Department of Fishery Products, Milk and Other Food of Animal Origin, Ministry of Rural Development and Food of Greece, Athens, Greece.; k Water Supply Planning and Support Operations Directorate, Athens Water Supply and Sewerage Company, Athens, Greece.; l Faculty of Medicine, University of Thessaly, Larissa, Greece.; m Ministry of Health, Athens, Greece.

## Abstract

Floods are among the most frequent and complex climate-related hazards, affecting health and health systems through infectious disease risks, water and chemical contamination, and disruption of essential services. Examining how international guidance reflects the operational realities of flood response, and how it supports countries in practice, is essential. While each event is unique, sharing experiences can strengthen preparedness and resilience. This paper draws lessons from an assessment of the response measures used during the 2023 floods in Greece. The coordination team rapidly adapted existing structures for infectious disease surveillance, vaccination and water quality monitoring to an emergency response of exceptional scale. To track flood-related diseases, the coordination team reconfigured syndromic surveillance tools initially designed for refugee camps, and compared proportional morbidity with baseline data to provide early warnings. The team also implemented multiplex polymerase chain reaction diagnostics and mobile vaccination campaigns targeting high-risk groups. Risk communication and community outreach combined repurposed materials with new flood-specific guidance. While there were no outbreaks of gastroenteritis, respiratory infections or mosquito-borne diseases, 45 leptospirosis cases and one fatal non-toxigenic *Vibrio cholerae* infection highlighted the need for sustained vigilance. The lessons learnt indicate that preparedness relies on flexible adaptation of existing systems, cross-governmental coordination and active community engagement. Gaps in global and regional guidance, especially regarding laboratory diagnosis, safe water restoration and chemical safety monitoring, complicate field decision-making. Strengthening international frameworks, embedding flood preparedness within climate adaptation strategies and applying One Health approaches are therefore critical to ensure health system resilience as flood events become increasingly frequent and severe.

## Résumé

Les inondations comptent parmi les risques climatiques les plus fréquents et les plus complexes. Elles ont des répercussions sur la santé et les systèmes de santé en raison des risques de maladies infectieuses, de la contamination de l’eau par des produits chimiques et de la perturbation des services essentiels. Il est crucial d’examiner la manière dont les directives internationales reflètent les réalités opérationnelles de la réaction aux inondations et dont elles soutiennent les pays en pratique. Même si chaque événement est unique, le partage d’expériences permet de renforcer le degré de préparation et la résilience. Le présent article tire les enseignements d’une évaluation des mesures d’intervention mises en œuvre lors des inondations de 2023 en Grèce. L’équipe de coordination a rapidement adapté les structures existantes de surveillance des maladies infectieuses, de vaccination et de contrôle de la qualité de l’eau afin de mettre en place une intervention d’urgence d’une ampleur exceptionnelle. Afin de suivre les maladies en lien avec les inondations, elle a reconfiguré les outils de surveillance syndromique initialement conçus pour les camps de réfugiés et a comparé la morbidité proportionnelle aux données de référence afin de fournir des alertes précoces. Elle a également mis en œuvre des diagnostics par réaction en chaîne par polymérase (PCR) multiplexe et des campagnes de vaccination mobiles ciblant les groupes à haut risque. La communication des risques et la sensibilisation des populations locales ont combiné la réutilisation de supports et de nouvelles directives spécifiques aux inondations. Malgré l’absence de signalement d’épidémie de gastro-entérite, d’infections respiratoires ou de maladies transmises par les moustiques, 45 cas de leptospirose et un cas mortel d’infection à *Vibrio cholerae* non toxigène ont souligné la nécessité d’une vigilance soutenue. Les enseignements tirés indiquent que la préparation repose sur une adaptation flexible des systèmes existants, une coordination intergouvernementale et une participation active des populations locales. Les lacunes dans les directives mondiales et régionales, notamment en matière de diagnostic en laboratoire, de rétablissement de l’approvisionnement en eau potable et de surveillance de la sécurité chimique, compliquent la prise de décision sur le terrain. Il est donc essentiel de renforcer les cadres internationaux, d’intégrer la préparation aux inondations dans les stratégies d’adaptation au changement climatique et d’appliquer les approches « One Health » afin de garantir la résilience des systèmes de santé, alors que les inondations se font plus fréquentes et plus graves.

## Resumen

Las inundaciones se encuentran entre los peligros relacionados con el clima más frecuentes y complejos. Además, afectan a la salud y a los sistemas de salud mediante riesgos de enfermedades infecciosas, contaminación del agua y por sustancias químicas, y la interrupción de servicios esenciales. Es esencial examinar en qué medida la orientación internacional refleja las realidades operativas de la respuesta a inundaciones y cómo apoya a los países en la práctica. Aunque cada evento es único, el intercambio de experiencias puede fortalecer la preparación y la resiliencia. En este artículo, se extraen lecciones de una evaluación de las medidas de respuesta aplicadas durante las inundaciones de 2023 en Grecia. El equipo de coordinación adaptó rápidamente las estructuras existentes de vigilancia de enfermedades infecciosas, vacunación y monitoreo de la calidad del agua a una respuesta de emergencia de escala excepcional. Para hacer seguimiento de las enfermedades relacionadas con las inundaciones, el equipo de coordinación reconfiguró herramientas de vigilancia sindrómica diseñadas inicialmente para campamentos de refugiados y comparó la morbilidad proporcional con datos basales para proporcionar alertas tempranas. El equipo también implementó diagnósticos mediante reacción en cadena de la polimerasa múltiplex y campañas móviles de vacunación dirigidas a grupos de alto riesgo. La comunicación de riesgos y la divulgación comunitaria combinaron materiales reutilizados con nuevas orientaciones específicas para inundaciones. Aunque no se registraron brotes de gastroenteritis, infecciones respiratorias ni enfermedades transmitidas por mosquitos, 45 casos de leptospirosis y un caso mortal de infección por *Vibrio cholerae* no toxigénico destacaron la necesidad de mantener una vigilancia sostenida. Las lecciones aprendidas indican que la preparación depende de la adaptación flexible de los sistemas existentes, la coordinación intergubernamental y la participación activa de la comunidad. Las lagunas en la orientación mundial y regional, especialmente en lo relativo al diagnóstico de laboratorio, el restablecimiento seguro del suministro de agua y el monitoreo de la seguridad química, complican la toma de decisiones sobre el terreno. Por consiguiente, reforzar los marcos internacionales, integrar la preparación ante inundaciones en las estrategias de adaptación al clima y aplicar enfoques de «Una sola salud» resultan fundamentales para asegurar la resiliencia de los sistemas de salud a medida que las inundaciones se vuelven cada vez más frecuentes y graves.

## ملخص

تُعد الفيضانات من أكثر المخاطر المناخية شيوعًا وتعقيدًا، إذ تؤثر على الصحة وأنظمة الرعاية الصحية من خلال مخاطر الأمراض المعدية، وتلوث المياه والمواد الكيميائية، وتعطيل الخدمات الأساسية. إن دراسة كيفية انعكاس التوجيهات الدولية على واقع الاستجابة للفيضانات، وكيفية دعمها للدول عمليًا، أمر بالغ الأهمية. ورغم أن كل حدث فريد من نوعه، فإن تبادل الخبرات يمكن أن يعزز الاستعداد والقدرة على الصمود. تستخلص هذه الورقة الدروس من تقييم التدابير المتخذة خلال فيضانات عام 2023 في اليونان. فقد قامت فرق التنسيق بسرعة بتكييف الهياكل القائمة لمراقبة الأمراض المعدية، والتطعيم، ورصد جودة المياه لتلبية استجابة طارئة واسعة النطاق. ولتتبع الأمراض المرتبطة بالفيضانات، أعادت فرق التنسيق تهيئة أدوات المراقبة المتلازمية التي صُممت في الأصل لمخيمات اللاجئين، وقارنت المعدلات النسبية للإصابة بالأمراض بالبيانات الأساسية لتوفير إنذارات مبكرة. كما نفذت الفرق تشخيصات بتقنية تفاعل البوليميراز المتعدد (PCR) وحملات تطعيم متنقلة تستهدف الفئات عالية الخطورة. جمعت جهود التواصل بشأن المخاطر والتوعية المجتمعية بين مواد معاد استخدامها وتوجيهات جديدة خاصة بالفيضانات. ورغم عدم حدوث حالات تفشي لالتهابات الجهاز الهضمي أو أمراض الجهاز التنفسي أو الأمراض المنقولة بالبعوض، فإن تسجيل 45 حالة من داء اللولبية النحيفة وحالة وفاة واحدة بسبب عدوى غير سامة ببكتيريا الكوليرا (Vibrio cholerae) أبرز الحاجة إلى اليقظة المستمرة. تشير الدروس المستفادة إلى أن الاستعداد يعتمد على التكيف المرن للأنظمة القائمة، والتنسيق بين مختلف الجهات الحكومية، والمشاركة الفعالة للمجتمع. كما أن الفجوات في التوجيهات العالمية والإقليمية، خصوصًا فيما يتعلق بالتشخيص المخبري، واستعادة المياه الآمنة، ورصد السلامة الكيميائية، تعقد عملية اتخاذ القرار الميداني. لذلك فإن تعزيز الأطر الدولية، ودمج الاستعداد للفيضانات ضمن استراتيجيات التكيف مع المناخ، وتطبيق نهج "الصحة الواحدة" يُعد أمرًا حاسمًا لضمان مرونة أنظمة الصحة مع تزايد تواتر وشدة أحداث الفيضانات.

## 摘要

洪灾现已成为 种频发且复杂的气候相关灾害，会引发传染病传播风险，造成水体和化学品污染及基本服务中断，进而对健康和卫生系统造成冲击。研究分析国际指南并核实其如何反映洪灾应对实操实际，及如何切实支持各国应对洪灾，这一点至关重要。虽然每次洪灾情况都各有不同，但是分享经验能够增强防备和抵御能力。本文通过评估希腊在 2023 年洪灾中所采用的应对措施，总结了经验教训。其协调小组快速调整已有的传染病监测、疫苗接种和水质监测结构，以启动超大规模的应急响应。为了追踪洪灾相关疾病，协调小组将最初设计用于难民营的症状监测工具进行了重新配置，并将发病率占比与基线数据对比，以提供早期预警。协调小组还实施了多重聚合酶链反应诊断，并针对高危人群开展了流动疫苗接种行动。通过开展风险沟通和社区外联工作，将经改造复用的宣传材料与针对洪灾的全新专项指导内容相结合。在此期间，虽然未爆发肠胃炎、呼吸道感染或蚊媒传染病，但是 45 例钩端螺旋体病和 例致命的非产毒型霍乱弧菌感染仍凸显了保持警惕的必要性。通过总结的经验教训可以得出，开展洪灾防备工作有赖于现有系统的灵活调整、跨政府部门的协调配合和社区的主动参与。由于全球性和区域性指南之间存在缺口，特别是在实验室诊断、安全供水恢复及化学品安全监测方面，这导致现场决策进 步复杂化。因此，随着洪灾日益频发和严重，完善国际相关框架、将洪灾防备工作纳入气候适应战略体系以及采用“同 健康”方法是确保卫生系统抗灾韧性的关键所在。

## Резюме

Наводнения это одна из самых частых и сложных угроз, связанных с изменением климата. Их влияние на здоровье и системы здравоохранения связано с рисками распространения инфекционных заболеваний, загрязнения воды и химического загрязнения, а также со сбоями в работе жизненно необходимых служб. Крайне важно изучать то, как международные руководства отражают оперативную реальность ликвидации последствий наводнений и как они поддерживают конкретные страны на практике. Хотя каждое такое событие уникально, обмен опытом может укрепить готовность и системную устойчивость. В данной статье обсуждаются уроки, извлеченные из оценки мер по ликвидации последствий наводнения в Греции в 2023 году. Координационная группа быстро адаптировала имеющиеся структуры надзора за инфекционными заболеваниями, группы вакцинирования и мониторинга качества воды для реагирования на чрезвычайную ситуацию исключительного масштаба. Чтобы отслеживать заболевания, связанные с наводнением, координационная группа переформатировала средства синдромного надзора, изначально разработанные для лагерей перемещенных лиц, и сравнивала пропорциональную заболеваемость с исходными данными для обеспечения раннего предупреждения. Группа также внедрила мультиплексную полимеразную цепную реакцию как средство диагностики и провела мобильные кампании вакцинирования с прицелом на группы высокого риска. Информирование о рисках и работа с общественностью сочетали адаптированные ранее материалы с новыми рекомендациями, специально разработанными для условий наводнений. Хотя вспышек гастроэнтерита, респираторных инфекций или инфекций, переносимых комарами, не наблюдалось, 45 случаев лептоспироза и одна смерть в результате инфекции нетоксикогенным штаммом *Vibrio cholerae* подчеркнули необходимость постоянной бдительности. Извлеченные уроки показывают, что процесс обеспечения готовности опирается на гибкую адаптацию имеющихся систем, координацию различных органов власти и активное вовлечение общественности. Недочеты в глобальных и региональных руководствах, особенно в отношении лабораторной диагностики, восстановления безопасности воды и мониторинга химической безопасности, осложняют принятие решений на местах. Укрепление международных рамочных структур, включение обеспечения готовности к наводнениям в стратегии адаптации к изменению климата и применение подхода «Единое здоровье» являются критически важными для обеспечения устойчивости систем здравоохранения в условиях более частых и серьезных случаев наводнения.

## Introduction

Climate change exerts widespread effects across all continents, influencing both human and animal populations.^1^ The most evident manifestation of climate change is the rising intensity and frequency of extreme weather events.^2^ Under a changing climate, heavy precipitation episodes are projected to occur with substantially greater intensity and up to nine times more often.^3^

Between 1998 and 2017, floods affected more than 2 billion people worldwide and comprised 44% (3254/7348) of all natural disasters from 2000 to 2019.^4^ Flooding has substantial impacts on health and is associated with acute, medium- and long-term effects on health and well-being.^5^ Floodwaters often contain contaminants that can cause irritation or infections, and these contaminants may remain in the environment long after the water has receded. Infectious disease outbreaks, such as those caused by vector-borne pathogens, can occur as breeding sites for some vectors increase.^5^ Moreover, floods often damage sewage systems, leading to the release of human and animal faecal waste into floodwaters and thereby increasing health risks.^5^ Flood events can destroy homes, businesses and personal property, disrupt power and water supplies, and limit access to essential public services. Finally, disruptions to water, sanitation, power, transport and communications during post-flood cleanup create conditions that increase the risk of infection.^6^ Therefore, comprehensive preparedness is crucial, enabling countries to prevent, detect and control the complex health risks that may emerge following flood events. 

## International policy frameworks

Several international and regional regulations, guidelines and frameworks are in place to support countries in flood preparedness. The *International health regulations (2005*) (IHR (2005)) mandate core surveillance and response capacities for public health risks with the potential of international spread,^7^ while the Sendai Framework for Disaster Risk Reduction 2015–2030 guides stakeholders to engage in multihazard risk reduction and to develop resilient health systems.^8^ The Paris Agreement commits Parties to climate adaptation and vulnerability reduction.^9^ World Health Organization (WHO) guidance further recommends multisectoral, whole-of-government and whole-of-society approaches to health emergencies and disasters.^10^ In the European Union (EU), the EU directive on the assessment and management of flood risks obliges Member States to map risks and prepare management plans,^11^ complemented by the Union Civil Protection Mechanism for cross-border coordination^12^ and the EU Climate Adaptation Strategy for mainstreaming resilience.^13^

WHO and public health agencies provide specific guidance for governments on flood response. For an effective response, WHO underscores rapid risk assessment, safe water and sanitation, vaccination, community engagement and system-wide preparedness through coordinated surveillance and diagnostic capacity.^14^^–^^16^ Furthermore, the United States Centers for Disease Control and Prevention provides detailed guidance on flood-related health risks, including cleanup, mould remediation, vector control and safe restoration of water supplies, while its Public Health Emergency Preparedness and Response Capabilities framework outlines readiness standards applicable to flood scenarios.^17^^,^^18^ The European Centre for Disease Prevention and Control (ECDC) supports EU Member States by issuing risk assessments during major flood events, focusing on infectious diseases such as leptospirosis, West Nile virus and gastrointestinal infections.^19^

However, whether this international guidance sufficiently aligns with the operational realities of flood response and provides the level of detail needed to support decision-making in the field remains to be determined. As each flood event presents unique challenges shaped by local vulnerabilities and capacities, systematically capturing and sharing experiences is essential to strengthen evidence-informed policies and ensure that future guidance reflects real-world needs. In this article, we describe the response measures and adaptations implemented during a flood event in Greece, and discuss strengths, operational gaps and lessons learnt from this response. For this report, we used aggregated and anonymized data, with ethical approval obtained from the University of Thessaly (07/06.02.2024). Issues related to rescue operations, mental health, provision of housing for displaced populations and management of animal carcasses are outside the scope of this article.

## Evidence in practice

### Context

In September 2023, the storm Daniel hit Bulgaria, Greece and Türkiye.^20^ Within 24 hours, between 5 and 6 September, specific areas of Greece received the equivalent of approximately 18 months of rainfall.^20^ Developing characteristics of a Mediterranean hurricane as it moved towards Libya, the storm resulted in the collapse of two Libyan dams, with subsequent flooding leading to thousands of deaths.^20^^,^^21^ In Greece, the estimated flooded area was 1150 km^2^, with the most affected areas along the Pineios River of the Thessalian plain (approximately 820 km^2^) in Greece.^22^ The flooding caused extensive damage to homes, primary health-care facilities, businesses, government buildings, schools, farms, road infrastructure, water supplies, electricity networks, wastewater treatment plants and cemeteries. An estimated 15% of the country’s annual agricultural yield was destroyed.^23^ During the first two weeks following the flooding, about 27 000 tons of untreated wastewater were discharged per day into the Pineios river (Mouchtouri VA, University of Thessaly, Greece, personal communication, 20 October 2023). Floodwaters swept away animal carcasses, vehicles, farm chemicals and untreated wastewater, threatening water, tourism and aquaculture in the Aegean Sea. Government reports estimated losses of more than 100 000 livestock and 130 000 fowl, representing 10% and 5% of domesticated animals, respectively.^24^

### Response

#### Coordination and governance

Greece had met its commitments to the EU by developing and sharing civil protection plans, with flood preparedness governance structures in place, including defined institutional responsibilities, coordination mechanisms and approved civil protection plans. However, the unprecedented scale of the flood required considerable adaptations to the already existing plan, as there were unforeseen challenges and detailed operational guidance from international sources was lacking for some needs. Within days of the flood, a multisectoral coordination centre was established through a new legislative act, bringing together central, regional and local authorities in line with WHO guidelines (Box 1).^10^ The centre was fully operational for three months, and the coordination team met daily to monitor emerging public health threats and coordinate response efforts. Building on the general plan for responding to emergencies, the coordination team developed a specific action plan by engaging relevant representatives, mapping previously unaddressed functions and updating contact details to ensure direct communication lines. The coordination centre issued daily and weekly reports to authorities, while the Hellenic health ministry informed the public through 40 announcements on water safety until December 2023, including guidance on bottled water use, and boiling advisories where water supplies were unsafe or damaged.

Box 1 **Entities participating in the multisectoral coordination team responding to the flooding caused by storm Daniel, Greece, 2023**

Ministry of Health; Ministry of Rural Development and Food; Ministry of Climate Crisis and Civil Protection; Ministry of Defence - Hellenic National Defence General Staff; Ministry of Environment and Energy; Hellenic Authority for Geological and Mineral Exploration; National Public Health Organization; National Reference Laboratories; Laboratory of Hygiene and Epidemiology; Peripheral Public Health Laboratory of Thessaly; 5th Regional Health Authority of Thessaly & Central Greece; Hellenic First Army (field army); Administration of the Region of Thessaly; Larissa General University Hospital; General Hospitals of Larissa, Volos, Trikala, Karditsa; Private Regional Hospital; 404 General Military Hospital of Larissa; General Directorates of Public Health and Social Care of Thessaly region and of regional units; Decentralized Administration of Thessaly and Central Greece; Water Directorate of Thessaly; Directorate of Veterinary Public Health; primary health-care centres; municipal water supply and sewerage enterprises; and water supply planning and support operations directorate.

Within the first 10 days, the National Public Health Organization and the Laboratory of Hygiene and Epidemiology at University of Thessaly conducted an internal rapid risk assessment of potential disease outbreaks. This assessment used the ECDC risk-calculation methods, adapted to the flood context.^25^ This assessment considered vulnerable groups, including older adults, people with underlying respiratory or cardiovascular diseases, people living with diabetes or immunosuppression, as well as Roma communities.

#### Surveillance

To create an enhanced syndromic surveillance system, members of the coordination team rapidly adapted the National Public Health Organization refugee camp scheme by focusing on gastroenteritis and respiratory tract infections, which were prioritized through the internal risk assessment. For laboratory confirmation of infections, serology, polymerase chain reaction (PCR) analyses, sequencing and sample referral to reference laboratories were made available.^26^ To strengthen diagnostics, 13 multiplex PCR machines, some relocated from unaffected areas, were deployed to hospitals and primary care facilities, enabling rapid detection of 22 gastrointestinal and 23 respiratory pathogens. 

The coordination team established a reporting system involving five hospitals, 21 health centres, a private clinic and a military hospital, which treated affected individuals and reported data to the team via an electronic platform. Using the data, the team analysed proportional morbidity using Shiny R (R Foundation, Vienna, Austria)^27^ and the Farrington method.^28^ To assess excess morbidity, the team compared hospitalization data with the previous four years.^29^ Health facilities received continuous communication about the surveillance procedures.

Through a webinar, the team also trained over 100 health workers in syndromic surveillance and use of diagnostic tools.

During the surveillance period, from 11 September to 31 December 2023, 3985 gastroenteritis cases and 19 492 respiratory tract infections were recorded (online repository).^30^ No outbreaks of gastroenteritis or respiratory infections were detected. Overall, 45 leptospirosis cases (6.5/100 000 population of the Thessaly region) were recorded, two of which were fatal. This outcome represented a marked increase compared to the 47 cases that were reported in Thessaly from 2004 to 2022.^31^ Of the 45 reported cases, 91% (41) occurred within the first five weeks following the flood event. Five *Leptospira interrogans* cases and 33 *L. kirschneri* cases were also reported. One fatal case of non-toxigenic *Vibrio cholerae* was confirmed. The number of West Nile virus cases remained within expected levels.

Among 422 gastroenteritis and 1205 respiratory samples tested by PCR, *Campylobacter*, Enteropathogenic *Escherichia coli*, norovirus, influenza A/H1N1–2009, rhinovirus/enterovirus and severe acute respiratory syndrome coronavirus 2 were the most common pathogens (online repository).^30^ Thirty-eight salmonellosis cases were identified, including one *Salmonella typhi*.

The only outbreak attributed to the floods was leptospirosis. The National Public Health Organization adapted leptospirosis prevention response by first using existing health education materials until flood-specific materials were developed. These flood-specific materials included guidance on leptospirosis prevention measures, an educational leaflet on leptospirosis and general guidance on the prevention of flood-related infections. Furthermore, mobile units of the National Public Health Organization conducted door-to-door campaigns and distributed personal protective equipment (4300 goggles, 25 000 suits, 1000 masks and 30 000 repellents) to affected and at-risk populations. They also provided messaging to promote early recognition of symptoms and timely health-care seeking for leptospirosis and other flood-related infections among affected populations, as well as training for students, parents, journalists, health workers, religious leaders and farmers.^32^

#### Water and environmental safety

The flood damaged potable water systems, including springs, wells and distribution networks. The absence of centralized mapping of the Thessaly region hindered rapid water source identification, while damaged roads further delayed risk assessment and corrective actions in highly affected areas. Water safety personnel from public services made on-site inspections of municipal water systems and tested for contamination. The coordination team established an emergency sampling programme, which covered wells, springs, tanks and distribution points. Existing organizational structures and professional networks for water sampling and testing were used, and external experts were invited to join the water safety group. A subgroup of water safety experts evaluated daily microbiological, physicochemical and field data, enabling the health ministry to issue timely advisories for residents. Adaptation of the advisories involved merging and jointly reviewing all results by members of the coordination team and supervisory authorities in daily assessments. Water safety experts in the coordination team highlighted that water safety plans based on WHO methods served as tools to strengthen restoration of damaged systems and guide corrective actions.^33^

Out of 5282 water samples, 65% (3413) were tested microbiologically and 73% (2495) of these met legal requirements for drinking water; 239 of 533 distribution systems were damaged and 56% (50/90) of sampled water sources showed faecal contamination, with most sources restored within two months. *Cryptosporidium* and *Giardia* species were not detected.

Monitoring of bathing water in seven different municipalities of the Region of Thessaly identified 25 of 206 samples exceeding the microbiological thresholds, with two beaches closed until June 2024. Shellfish production near the Pineios delta was suspended as a precaution, but 51 shellfish samples met EU microbiological standards.

Prevention of vector-borne diseases was strengthened after the flooding through intensified surveillance of West Nile virus transmission in high-risk areas, with over 55 000 adult mosquitoes captured. Of the 1117 *Culex pipiens* pools, 13 (1.2%) were West Nile virus-positive. Members of the coordination team and National Public Health Organization implemented risk-based mosquito control, which included larviciding, adulticiding, and ultra-low volume spraying.^34^ Despite increased breeding sites, West Nile virus disease notifications remained below expected levels for the area and period, likely reflecting the impact of coordinated and targeted vector control.

#### Vaccination and awareness 

Vaccination protocols were expanded to reach high-risk groups through health-promoting mobile units staffed by nurses and drivers. Following rapid risk assessment, Hepatitis A and tetanus vaccination was prioritized for Roma communities and first responders, with 188 and 2524 doses administered, respectively; no tetanus cases or Hepatitis A outbreak occurred. 

Adaptation also included rapid preparation of flood-specific communication plans and guidelines for affected communities, schools and accommodation facilities for displaced people. Public health promotion relied first on existing routine campaigns and other emergency materials until these were repurposed for flood-specific risk communication. The mobile units of the National Public Health Organization conducted 39 door-to-door campaigns, distributed 11 000 leaflets and 300 posters on leptospirosis and West Nile virus, and targeted these interventions in communities where stagnant water existed.

#### Guidance

Early in the response, limited published evidence and few comprehensive international guidelines and tools were available.^35^^,^^36^ The National Public Health Organization adapted some existing guidelines, such as those covering leptospirosis, West Nile virus infection and foodborne disease prevention, while eight guidelines had to be newly developed, as international guidance was not always specific. These new guidelines covered disease prevention, cleanup, water safety, mosquito protection and school reopening. Guidelines were published on the websites of the National Public Health Organization and the health ministry. Leading figures made formal announcements of the publications to authorities and affected populations. The adaptation of guidelines relied on repurposing existing tools. 

## Lessons learnt

The response to the flooding in Greece provided valuable lessons, showing that resilience and an effective response depend on sustained investment in cross-governmental coordination mechanisms that can be rapidly activated during emergencies.^10^ Furthermore, preparedness planning must anticipate that mitigation measures will be implemented under conditions of widespread disruption, including loss of electricity, telecommunications, water, food supplies, and waste management services and health facilities.^37^ The establishment of a multisectoral coordination centre facilitated timely situational mapping, risk prioritization and communication between diverse actors, enabling rapid surveillance, environmental sampling and targeted interventions. During the emergency, the defence ministry and general staff members of the national defence played a pivotal role by conducting on-site assessments, demonstrating the value of civil–military cooperation in crisis management.^38^ These experiences confirm that joint structures and interoperable systems across health, civil protection, defence, infrastructure and academic institutions are essential to ensure continuity of operations during a disaster.^39^ Notably, restoration of critical infrastructure needs to proceed in parallel with immediate infectious disease prevention measures to reduce secondary health impacts.^40^

Considering that no outbreaks of gastroenteritis, respiratory infections or mosquito-borne diseases occurred, and that hospitalizations were lower than in the previous four years, the response contributed to effectively reducing post-flood health risks. However, leptospirosis diagnosis proved challenging as laboratory capacity was established primarily for *L. interrogans*, while most cases were caused by *L. kirschneri*. If the circulation of *L. kirschneri* in local rodent populations had been known, diagnostic preparedness could have been strengthened. This challenge underlines the importance of a One Health approach to preparedness efforts, integrating human, animal and environmental surveillance for a more effective anticipation of zoonotic risks. 

Although regulatory frameworks for routine monitoring of drinking water, bathing waters, soil and aquaculture products exist in many countries, detailed protocols for post-flood contexts remain limited. Flooding can cause contamination with pathogens, chemicals and debris, yet little harmonized guidance exists on decision-making criteria for safely reopening potable water supplies, bathing waters or reintroducing aquaculture products.^41^ The coordination team considered the following criteria: (i) residual chlorine levels were within regulatory limits and remained stable over time; (ii) two consecutive water samples comply with microbiological quality standards; and (iii) a satisfactory sanitary inspection of the water supply system. Soil contamination, which may affect agriculture and food safety, is even less consistently addressed. In the absence of specific post-flood standards, local authorities may face uncertainty, balancing timely recovery with public health protection. Evidence from environmental health research emphasizes that flood-related contamination by pesticides, heavy metals and microplastics remains under-studied, with few documented response measures despite recognized risks.^41^ Hence, dual risk management strategies are needed that address both flooding and chemical hazards.^41^ These findings align with our assessment, showing that while adaptation of existing structures, plans, guidelines, surveillance systems and authorities is feasible and effective, more systematic integration of climate and environmental health into preparedness planning remains essential. If comprehensive water safety plans were fully implemented before flooding, restoration would be better facilitated, as detailed mapping of water sources, distribution networks and corrective actions would support faster decision-making. Developing context-adapted and internationally recognized protocols for post-flood environmental safety assessments could therefore strengthen consistency and resilience in public health practice.

Flood emergencies rarely allow new surveillance systems to be created from scratch; instead, existing structures must be rapidly adapted. In this emergency, the refugee camp syndromic surveillance system was reconfigured for daily flood-related infectious disease reporting, and a new data platform was developed.^42^ These experiences highlight the need for national systems to maintain capacity for ad hoc syndromic and laboratory surveillance, supported by trained staff and integrated within climate change adaptation strategies.^37^^,^^43^

Adaptations, including expanded vaccination, emergency sampling and expedited testing of water systems, repurposing risk communication tools, distribution of personal protective equipment and flood-specific public guidance, highlight the strength of having pre-existing systems for an effective response. However, preparedness planning for unprecedented disruption needs to include adaptation processes that embed flexibility and align national protocols with international standards.

Our findings show that education and awareness initiatives for health workers were essential to strengthen diagnostic and surveillance capacity, underscoring the importance of embedding climate and flood-related health risks into professional training to improve preparedness and resilience. Other studies address the need to integrate climate change as a health threat into health system preparedness.^44^^,^^45^

## Recommendations

Insights gained during the response to the complex flood event can guide preparedness and resilience planning at global, national and local levels.

### Global level

At the global level, important frameworks already guide preparedness efforts, including *IHR (2005)*, the Sendai Framework and the Paris Agreement, as well as subject-specific guidance on water and sanitation, food and prevention of diseases. While these frameworks establish essential obligations and general guidance, they are dispersed across multiple sources and may not always provide sufficient operational detail on flood-related health challenges, such as criteria for safely reopening water supplies, laboratory diagnosis of leptospirosis caused by different species, or monitoring chemical indicators in soil, bathing waters and aquaculture.^7^^–^^9^ The WHO emergency response framework emphasizes the value of multisectoral coordination, yet many practical decisions during flood events remain at the discretion of national authorities, often without harmonized standards.^10^

The diversity of flood risks, shaped by differences in geography, epidemiology, population density and environmental conditions, makes the development of universal protocols challenging.^35^^,^^36^ Nonetheless, experiences from recent events demonstrate that existing systems can be adapted effectively for flood-related infectious disease monitoring.^42^ Building on these lessons, global guidance could be strengthened by providing more detailed operational protocols that support both developed and developing countries in implementing timely and evidence-based measures.

In addition, integrating One Health approaches is critical, given the zoonotic dimension of many flood-related risks. Establishing systematic mechanisms for knowledge exchange across countries would help ensure that lessons from diverse flood contexts are translated into evolving global policy and practice.

Finally, as studies have identified gaps in addressing the safety of health workers during climate-related events,^39^ evidence-based recommendations are needed to ensure that workers are safe when responding to an emergency. 

### National level

At the national level, flood preparedness should be integrated within broader climate change adaptation strategies, with health resilience recognized as a cross-sectoral priority.^9^ Strengthening diagnostic and laboratory capacity is essential for rapid detection of flood-related infectious diseases, supported by scenario planning that considers known zoonotic threats in local animal populations. Incorporating a One Health perspective into preparedness planning can improve anticipation of risks and ensure diagnostic readiness.

Strengthened national water safety plans, aligned with WHO methods, can provide clear protocols for emergency sampling, phased restoration of supplies and transparent risk communication with the public.^10^ Beyond microbial testing, national standards should also address chemical indicators for aquaculture and bathing waters, reflecting gaps highlighted by recent flood events.

Finally, effective preparedness depends on formalized cross-governmental coordination. Engagement of ministries of health, civil protection, environment, agriculture and defence enhances both early risk assessment and operational response. Evidence from recent experience has shown that such multisectoral cooperation facilitates situational mapping, prioritization of risks and rapid implementation of protective measures, thereby strengthening overall system resilience.^10^^,^^38^

### Local level

At the local level, community engagement is central to effective flood preparedness. Populations should be actively involved in civil protection planning to ensure that strategies reflect local realities and capacities. Climate change health literacy must go beyond general awareness, incorporating participatory learning for health workers, school students (aged 6–18 years), journalists and farmers, enabling these groups to act as multipliers of preparedness knowledge.^44^^,^^45^

Local health services should be equipped with flexible surveillance tools, vaccination mechanisms that enable rapid distribution, and communication materials to support a swift response during floods. Building partnerships between responding authorities and municipalities, schools, farmer associations and religious leaders can further strengthen trust and encourage the uptake of protective measures.^44^ By integrating community knowledge with national and global frameworks, preparedness can become more responsive, context-specific and sustainable, enhancing resilience in the face of climate-related disasters.

## Conclusion

Floods represent one of the most complex climate-related hazards, generating cascading consequences for public health through infectious disease risks, water and chemical contamination, and disruption of essential services. Because every flood is unique, shaped by geography, infrastructure and epidemiology, the systematic sharing of experiences is essential to refine evidence-informed policy. Experience from recent large-scale floods shows that public health systems can adapt existing structures rapidly, yet important gaps remain in international guidance and national preparedness. By embedding climate adaptation strategies – supported by investments in laboratories, surveillance systems and formalized cross-governmental coordination – in national-level preparedness plans, countries can strengthen their capacity to anticipate, withstand and recover from future flood events. Recognizing local communities as active partners in preparedness planning is also important for an effective and context-appropriate response. Furthermore, a One Health approach, integrating human, animal and environmental health, can guide preparedness and response to more effectively address zoonotic threats. Ultimately, flood preparedness is not only a technical imperative but also a political and social commitment to protect populations from the growing health impacts of climate change. 
